# *Legionella pneumophila* in Municipal Shower Systems in Stavanger, Norway; A Longitudinal Surveillance Study Using Whole Genome Sequencing in Risk Management

**DOI:** 10.3390/microorganisms10030536

**Published:** 2022-02-28

**Authors:** Anne Vatland Krøvel, Eva Bernhoff, Elin Austerheim, Markus André Soma, Monica Regine Romstad, Iren Høyland Löhr

**Affiliations:** 1 NORCE—Norwegian Research Centre, Environment Department, Professor Olav Hansenssvei 15, N-4021 Stavanger, Norway; elau@norceresearch.no; 2 National Reference Laboratory for Legionella, Department of Medical Microbiology, Stavanger University Hospital, P.O. Box 8100, N-4068 Stavanger, Norway; eva.bernhoff@sus.no (E.B.); markus.andre.soma@sus.no (M.A.S.); monica.regine.romstad@sus.no (M.R.R.); iren.hoyland.lohr@sus.no (I.H.L.)

**Keywords:** *Legionella pneumophila*, surveillance, sequence types, municipal, shower systems, geographical clustering, WGS, source investigation

## Abstract

Following an incidence of Legionnaires disease (LD) in 2007, where a municipal shower system was the likely source of infection, Stavanger municipality initiated a surveillance program for *Legionella* as part of establishing internal risk evaluation and prevention routines. More than 250 shower systems were examined for cultivatable *Legionella pneumophila.* The prevalence and diversity of serogroups (sg) and sequence types (STs) of *L. pneumophila* were mapped using available typing techniques over a period of more than 10 years (2010–2021). The surveillance showed an overall reduction in the *L. pneumophila* colonisation rate in municipal systems from 11 to 4.5% following prevention measures during the period, with the highest colonisation rate in complex systems (e.g., larger nursing homes and sports complexes). Further, an approximately even distribution between sg1 and 2–14 was seen. Whole genome sequencing (WGS) revealed that only a limited number of STs were detected, and they were consistent at specific locations over time. This study showed that environmental surveillance data in combination with available typing techniques and WGS can give the municipality a better tool for risk management and an overview of ST distributions that can be a valuable asset in future source investigations.

## 1. Introduction

The *Legionella* bacteria can cause a severe and potentially fatal form of pneumonia called Legionnaires’ disease (LD) [1]. When the bacteria colonise and multiply in man-made systems with favourable conditions for growth, it may pose a threat to human health through inhalation of bacteria-contaminated aerosols [1,2]. *Legionella* is an opportunistic pathogen and the elderly or persons with compromised immune systems are at a greater risk of being infected [1]. 

There are more than 60 known species of *Legionella* with varying pathogenicity [3]. *Legionella pneumophila* has been implicated in at least 90% of the reported LD cases (reviewed in [4,5]). *L. pneumophila* can be subtyped into at least 15 different serogroups (sg) based on surface molecules and into different sequence types (STs), of which there are currently nearly 3000, based on a selected set of seven genes [6,7,8]. Sequence-based typing (SBT) is crucial for source investigations and epidemiological studies of *L. pneumophila.* Although the SBT performed by Sanger sequencing is still considered the gold standard, more recently, whole genome sequencing (WGS) has become the method of choice providing both ST type and superior information about genetic relatedness and is currently indispensable in outbreak investigations [9,10]. 

In Norway there have been two major outbreaks of LD. The first outbreak occurred in 2001 in Stavanger (Norway’s 4th largest city, located on the southwest coast), with 28 infected and 7 fatalities [11]. The source of this outbreak was the cooling tower of a hotel. The second outbreak occurred in 2005 in the southeast of Norway with 56 infected and 10 fatalities [12]. Here the source was identified as an air scrubber at an industrial plant. Following these incidents, temporary guidelines for the prevention of *Legionella* transferred from aerosols were implemented in Norway in 2005 along with the establishment of National Reference Laboratories for *Legionella* in Stavanger and Oslo. In 2008, the current regulations, which state that owners of buildings open to the public must build and maintain aerosol-producing devices in order to prevent exposure to *Legionella,* were implemented [13,14]. Prediction of the risk of infection by *Legionella* from water systems is based on multiple factors and can be challenging. The responsibility placed on building owners by the Norwegian regulations from 2008 was substantial, and with limited resources available to fulfil all the tasks and responsibilities in the public sector, a more targeted and cost-effective prevention routine based on risk assessment was required. 

In Stavanger, the only known case of LD where the likely source was a municipal shower system was reported in 2007. Following the incident it was stated that municipal prevention routines for *Legionella* were not in place [15]. A preliminary set of prevention routines was established, but when evaluated in 2009 they were found to be insufficient and cost-prohibitive [15]. As part of the development of new surveillance routines, the municipality of Stavanger initiated a surveillance program that included nearly all municipal shower systems and separated them into three risk categories based on *L. pneumophila* sg and system user groups. Different sgs have been connected to different levels of risk of LD, and the incorporation of such knowledge into the risk assessment was recommended [16]. The overall aims for these new surveillance routines were to establish a cost-effective, targeted prevention routine, as well as differentiating the sampling routines of municipal shower systems and focusing the limited municipal resources for sampling and treatment to the shower systems with the highest risk for LD. 

Here we report the prevalence and strain characteristics of *L. pneumophila* in shower systems detected through the municipal surveillance program in Stavanger from 2010–2021. We evaluate the success of the surveillance and targeted strategy implemented in 2012 to reduce *L. pneumophila* colonisation. In addition, we investigate whether the introduction of WGS provided data valuable for the surveillance, risk assessment, and potential source investigation of future LD cases. Since sampling was performed in shower systems in use, this data set gave a unique opportunity to study *Legionella* colonisation in authentic live systems over time.

## 2. Materials and Methods

### 2.1. Surveillance Program

The surveillance program for *Legionella* in Stavanger municipality was initiated by a two-year pilot study from January 2010 to December 2011 [15]. Based on the pilot study, an expanded surveillance program for *Legionella* was initiated from January 2012, including all identified municipal buildings containing a shower system (*n* = 256). The municipal buildings were divided into six groups according to use of the building: schools (S) (*n* = 49), nursing homes (H) (*n* = 14), sports complexes (SC) (*n* = 37), kindergartens (K) (*n* = 61), council homes (CH) (*n* = 49), and others (offices, district houses, and preparedness buildings) (O), (*n* = 46). The municipal buildings were separated into three risk categories with different sampling frequencies based on the known user groups/access to the system and incidence of *L. pneumophila* sg; see Table 1.

The risk category could change depending on the culture results or changes in the user groups. The municipal internal guidelines are open for interpretation when it comes to sampling frequency. The sample frequencies used during the project period are listed in the fourth column in Table 1.

Nursing homes were all classified as risk category 1. Schools and large sports complexes were placed in risk category 2. Risk category 3 included the majority of kindergartens, smaller sports complexes, council houses, and the buildings in the “other” category.

During the surveillance period, extensive measures were taken to reduce the level of *L. pneumophila*, especially in sg1-colonised systems. The measures are listed in Table 2. The scope of this article was to evaluate the strategy of targeting high-risk systems rather than the measures themselves, therefore the specific measures are not further described. Actions were based on risk evaluation, where sg1 was evaluated as more high-risk than sg2–14, and 10 cfu/mL was the action limit chosen by the municipality.

The municipal building portfolio is constantly changing and overall has grown over the more than 10-year project period. Some of the buildings have been demolished, taken out of use, or sold and new buildings have been acquired. The full surveillance program, when initiated in January 2012, counted 256 shower systems. If a building had more than one shower system with separate heaters, each system was counted separately. The number of shower systems under surveillance in December 2021 was 311 and included, to the best of our knowledge, all municipal shower systems. The distribution of systems in building categories were S (*n* = 50), H (*n* = 13), SC (*n* = 39), K (*n* = 72), CH (*n* = 53), and O (*n* = 85).

### 2.2. Sample Collection

The municipal surveillance program in Stavanger (including the pilot) has been ongoing since 2010, and more than 24,000 water samples have been included. In January 2020, several smaller neighbouring municipalities were merged into Stavanger municipality. We report on the shower systems of the original Stavanger municipality.

In this study, a selection of *L. pneumophila* isolates from the municipal surveillance program was included (*n* = 43) for further characterisation. Isolates were divided into two groups “Initial” (I, *n* = 28) and “Current” (C, *n* = 15). The “Initial” group consisted of the first isolates from all culture-positive systems detected in the pilot (2010–2011) and in the extended surveillance program in the following 2 years (2012–2014). The “Current” group was comprised of the latest isolates from all the culture-positive systems isolated in 2021, including two systems that were not culture-positive in the initial round, as well as one 2017 sample from one of the “new” systems. Due to the ongoing COVID-19 pandemic, planned sampling in 2020/2021 for the non-culture-positive systems (mainly risk category 3 systems), was postponed. Since sampling frequencies vary for different building categories, see Table 1, the latest results from these systems are from the period of 2016–2019.

Sampling of the shower water was conducted as described by [15]. Water samples from a number of selected sample points were analysed for each shower system. The number of sample points per system varied with size and complexity. Each sample point was sampled both pre- and post-flush. If either or both samples were culture-positive, the sample point was marked as positive. 

### 2.3. Culture Analysis

Water samples were cultivated and enumerated in cfu/mL as described in [15]. A simplified method based on the standard ISO 11731:1998 (E), with a detection limit of 5 cfu/mL, was used. In short, 200 µL of water was dripped directly on selective GVPC media (Glycin, Vancomycin, Polymyxin B, and Cycloheximidine) in triplicates and allowed to dry before incubation at 37 °C for 3–10 days. 

The recommended action limits for *Legionella* in a system varies among countries (0, 1–10 cfu/mL), and in the Norwegian guidelines no action limits are currently given [1,14,17,18,19]. For surveillance purposes in the municipal shower systems, the detection limit of 5 cfu/mL was considered sufficient [15].

The morphology of the colonies cultivated from the water samples from various sample points in the municipal shower systems were evaluated visually and under the microscope, and *Legionella* was confirmed by agglutination as either *L. pneumophila* sg1, *L. pneumophila* sg2–14, or *Legionella* spp. (Legionella Latex test, Oxoid). 

A shower system was considered positive if any of the sample points were culture-positive for *Legionella*. If 30% or more of the tested sample points in a system were culture-positive, the system was considered highly colonised [20,21].

Isolates were referred to the National Reference Laboratory for *Legionella* at the Department of Medical Microbiology, Stavanger University Hospital (SUS), Norway, for confirmation and subtyping.

### 2.4. Serotyping 

Colonies identified as *L. pneumophila* were confirmed at the National Reference Laboratory for *Legionella* by agglutination (Legionella Latex test, Oxoid). The serotyping by agglutination for the initial 28 isolates were supplemented by serotyping sg 2–14 into specific sg by the Dresden panel of monoclonal antibodies [16,22]. 

### 2.5. Sequence-Based Typing 

Sanger sequencing was the method routinely used at the National Reference Laboratory for *Legionella* for SBT until August 2019, when it was replaced by WGS. The 28 isolates included in the initial round were sequence-typed using Sanger sequencing according to the protocol of the European working group of *Legionella* infections (EWGLI) [6,7]. 

### 2.6. Whole Genome Sequencing

All *L. pneumophila* isolates, (*n* = 43; I, *n* = 28; C, *n* = 15) from culture-positive shower systems included in this project were whole-genome sequenced. 

DNA was extracted using MagNA Pure 96 with the Pathogen Universal 200 4.0 purification protocol (Roche Applied Science, Penzberg, Germany). Genomic libraries were prepared using Illumina Nextera DNA Flex library prep and were sequenced using the Illumina MiSeq system and the Illumina MiSeq Reagent Kit V3 (600 cycle) to obtain 2 × 300 bp paired end reads. 

For the reference genomes, in addition, a DNA library for nanopore sequencing was prepared and barcoded with the Ligation sequencing kit (SQK-LSK109) and the Native Barcoding Expansion 1–12 (EXP-NBD104) (Oxford Nanopore Technologies (ONT), Oxford, UK), then loaded onto a R9.4.1 MinION flow cell (FLO-MIN106D) and sequenced on the ONT GridION device (GRD-X5G003).

### 2.7. Assembly and Quality Control

TrimGalore v0.6.7 [23] was used to trim the short reads. De novo assembly was then performed with Unicycler v0.4.8 [24], which uses SPAdes v3.13.0 [25] and Pilon v1.23 [26]. FastQC v0.11.9 [27] and QUAST v.5.1.0rc1 [28] were used for quality control. The following quality criteria needed to be fulfilled for the sequence to be accepted: average read depth > 30×, number of reads > 100,000, number of contigs < 400, largest contig > 20,000 bp, N50 > 30,000 bp, and L50 < 30. GC content and total length were checked to ensure that they were in line with *L. pneumophila*.

STs of isolates from the assembled and quality checked WGS data were inferred using legsta V.0.5.1 [29].

### 2.8. Hybrid Assembly and Annotation of Reference Genomes

Guppy Basecalling Software v.5.0.16 [30] in super-accurate basecalling mode was used to basecall and de-multiplex long reads of 4 *L. pneumophila* isolates, which were further subjected to quality filtering using Filtlong v.0.2.0 [24], where reads shorter than 1 kbp were removed and 10% of the lowest quality reads were excluded. Hybrid assembly with short reads was performed with Unicycler v.0.4.8 [24] using SPAdes v3.13.0 and Pilon v.1.24 [26], followed by annotation using Rast v.2.0 [31] with default settings and the RASTtk annotation scheme. 

### 2.9. Phylogenetic Analysis

Illumina short reads of the 28 initial *L. pneumophila* isolates were mapped to the reference genome *L. pneumophila subs. pneumophila* str. Philadelphia-1 (accession: NC_002942.5), using RedDog v.1beta.11 [24], utilizing Bowtie2 v.2.2.9 [32] and SAMtools v.1.3.1 [33] to generate a core chromosomal single nucleotide polymorphism (SNP) alignment. Four additional ST-specific alignments of genomes belonging to ST1 (*n* = 5), ST154 *(n* = 8), ST574 (*n* = 7), and ST1341 (*n* = 12) were also created with RedDog by mapping the genomes to their corresponding hybrid-assembled reference genomes. Default parameters were used in all RedDog alignments, as described previously [34]. For the ST-specific alignments, recombinations were filtered using Gubbins v.2.4.1 [35] with the convergence method ‘weighted Robinson–Foulds’. The generated alignments from Gubbins were further processed as input to snp-dists v.0.7.0 [29] for calculating SNP distance matrices. The same alignments were also passed to RAxML v.8.2.12 [36] with the GTRGAMMA model to infer the maximum likelihood (ML) phylogeny, after which the best-scoring ML tree was selected based on five independent runs. For the 28 isolates mapped to the reference strain Philadelphia-1 the RedDog alignment file was used as the input for snp-dists and RAxML. The phylogenetic trees were visualized in R v.4.0.5 [4] using the ggtree package [37].

### 2.10. Data Availability

The 43 short-read and 4 long-read sequence files were deposited in the European Nucleotide Archive under BioProject PRJEB50383 (see Supplementary Tables S1 and S2). 

## 3. Results

### 3.1. Prevalence of *L. pneumophila* in Shower Systems in Different Municipal Building Categories

**Figure 1:**
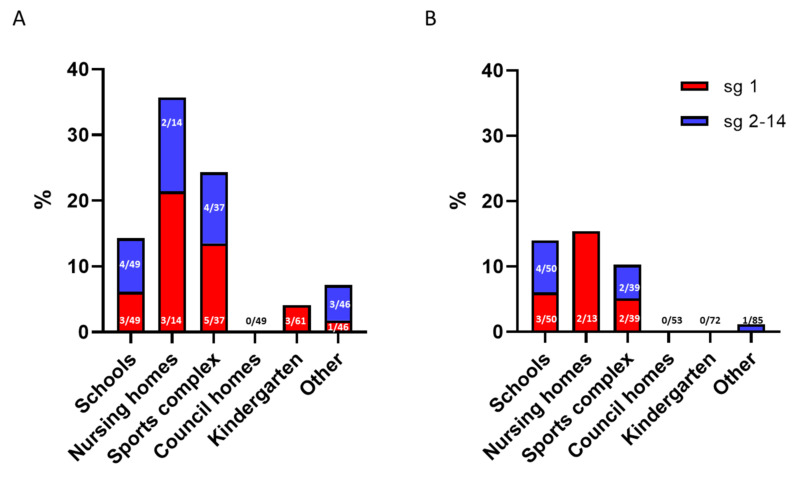
Relative prevalence of *L. pneumophila* in shower systems in different municipal building types. The *y*-axis indicates the percentage of *L. pneumophila* culture-positive shower systems, and the *x*-axis indicates the different municipal building categories. (**A**) The initial round of sampling. (**B**) The current round of sampling. Red bars represent *L. pneumophila* sg1, and blue bars represent *L. pneumophila* sg2–14. The numbers on the bars indicate the actual number of culture-positive shower systems compared to the total number of shower systems tested.

During the initial sampling, approximately 11% (28/256) of the municipal shower systems were culture-positive for *L. pneumophila*. The prevalence of *L. pneumophila* varied between 0% and 36% positive systems among the six different building categories. Buildings with large complex hot water systems, such as schools, nursing homes and sports complexes showed the highest relative colonisation rates of 15% (7/49), 36% (5/14), and 22% (9/37), respectively, see Figure 1A. 

At the end of the project period, there was a total reduction in the number of culture-positive systems to 14 of 311 systems, reflecting both a decrease in the number of culture-positive shower systems and an increase in the total number of shower systems under surveillance. Still, the highest colonisation rates were detected in the large and complex systems: schools 13% (7/50), nursing homes 16% (2/13), and sports complexes 10% (4/39), see Figure 1B.

A reduction in the number of culture-positive systems during the project period was observed in sports complexes *(n* = 5), nursing homes (*n* = 3), kindergartens (*n* = 3), and “other” buildings (*n* = 3). 

### 3.2. Culture Status and Characterisation of *L. pneumophila* Isolates

Serotyping of cultivatable *L. pneumophila* from municipal shower systems in the initial sampling round showed a distribution between sg1 and sg2–14 of 54% (15) and 46% (13), respectively, see Table 2. Currently, the distribution of sg1 and sg 2–14 is 50% (7) and 50% (7), respectively. The agglutination tests gave consistent results for multiple sampling points within one sampling and for consecutive samplings for the different shower systems.

SBT identified 10 different STs in total, with the majority belonging to ST1, ST154, ST574, ST1341, and ST1361, see Table 2. There was consistency between sg-typing and SBT for all STs except ST1341 and ST1361, where two different sgs were detected within the same ST. For all culture-positive systems except one (H5), *L. pneumophila* isolates with the same sg and ST were detected at both the initial and current sampling. Four of the systems were also co-colonised with a lower relative fraction of *Legionella anisa*. 

Initially 90% (25/28) of the culture-positive shower systems were considered highly colonised (>30% positive sampling points). During the project period, systems positive for *L. pneumophila* were subjected to extensive preventive measures towards *Legionella*, see Table 2. In general, more effort was made to decontaminate systems culture-positive for sg1 due to their higher risk (risk category 1) than systems culture-positive for sg2–14 (risk category 2). This resulted in a higher number of decontaminated systems for sg1 (5 systems) than sg2–14 (0 systems), a greater reduction in the number of highly colonised systems (14/16 to 2/12 vs. 13/14 to 7/7 for sg1 and sg2–14, respectively) and a higher reduction in the bacteria level for sg1 systems (all 12 current systems reduced to 10 cfu/mL or less) compared to sg2–14 systems (5/7 current systems, more than 10 cfu/mL detected) over the project period. 

The current status is that five (H3, S3, SC3, SC6, and SC7) of the 28 initially colonised systems have been successfully decontaminated for *L. pneumophila*. One system (SC4) has successfully decontaminated shower water due to end-point filtration, and two additional systems (S8 and O5) have become colonised by *L. pneumophila*, resulting in a current total of 14 culture-positive systems. Eleven of the initial systems are no longer part of the municipal building portfolio. Of the culture-positive systems, 10 are still considered highly colonised.

### 3.3. Geographical Distribution and Phylogenetic Analyses of *L. pneumophila* Isolated from Municipal Shower Systems

**Figure 2:**
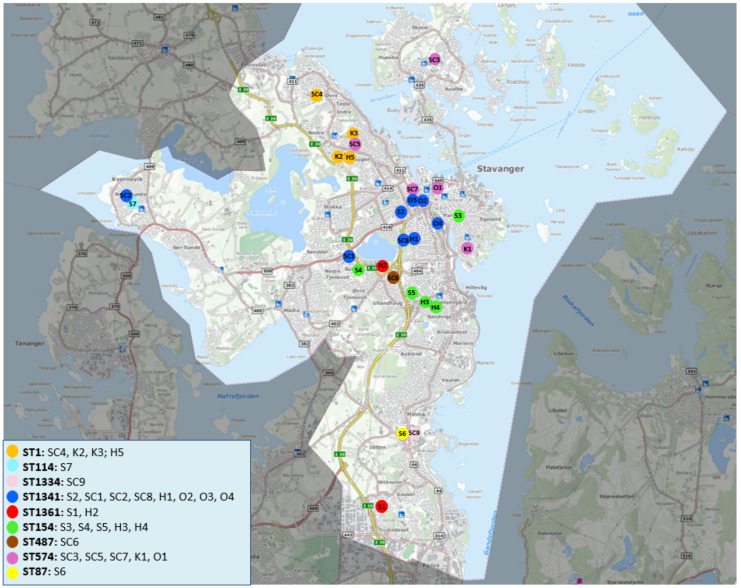
Geographical distribution of *L. pneumophila* STs in shower systems in the Stavanger municipality in the initial round. Each dot represents a municipal shower system containing *L. pneumophila*. Each ST is indicated with a distinct colour. The building code is the same as presented in Table 2, the capital letters represent the building type (SC = Sports Complexes, S = Schools, H = Nursing homes, K = Kindergarten, and O = Others) and the numbers represent the specific system. The highlighted section indicates the project area.

Of the 28 initial culture-positive shower systems, 21 systems (75%) contained 1 of the 4 sequence types ST1, ST154, ST574, or ST1341, see Table 2. These four main STs seem to be geographically distributed in clusters, see Figure 2. ST1, ST154, and ST1341 cluster quite closely together. ST574 appears to form a looser cluster, covering a larger area that is intertwined with the other identified clusters. Interestingly, in system H5, ST1 and ST574 were identified in the initial and current sampling rounds, respectively, and this building is positioned in a geographical area containing both STs. The ST1341 isolates have two different sgs, sg3 and sg6. This difference is also reflected in the map, where the sg3 system (SC2) is located far away from the sg6 isolates that group together (S2, SC1, SC8, H1, O2, O3, and O4). 

**Figure 3:**
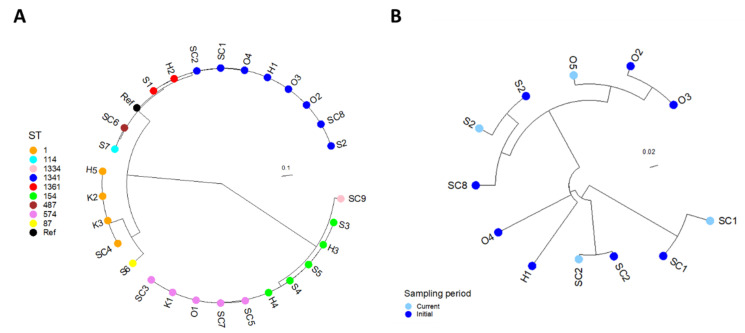
Maximum likelihood phylogeny of (**A**) the 28 initial *Legionella* culture-positive systems in Stavanger municipality and (**B**), the ST1341 isolates sampled at two different time periods. The isolates in both (**A**,**B**) are named according to the building code from which they were sampled. In (**A**), the different STs are represented by coloured nodes, where isolates of the same ST are seen clustering together, indicating relatedness. In (**B**), the phylogenetic relationships between the eight initial isolates of ST1341 (dark blue) and the four current isolates of the same ST (light blue) are inferred. Isolates that were sampled from the same building at two different time periods (initial and current), for example S2, cluster together, confirming that these isolates remain highly related and stable over time.

The phylogenetic analysis of the 28 initial isolates showed that isolates with the same ST cluster together, see Figure 3A. Furthermore, the STs with the most similar allelic SBT profiles (listed in Table 3) cluster together, for instance ST574/ST154/ST1334 and ST1341/ST1361.

For the four STs not assigned to a geographic cluster (ST59, 87, 114, and 1361), the initial and current isolates showed less than three SNP differences (Supplementary Tables S4–S6 and S9). ST-specific SNP analyses for the clusters ST1, ST154, and ST574, showed that the isolates had less than 15 SNP differences within the clusters and, thus, were highly related (Supplementary Tables S3, S7 and S8). The distance matrix for ST1341 showed up to 40 SNP differences and the isolates were subjected to an ST-specific phylogenetic analysis, see Figure 3B. From the ST-specific tree, we see that isolates from the same system are more similar than from different systems.

The phylogenetic analyses confirm that the isolates of the same ST are very similar and close to clonal, that they group according to their SBT profiles, and that they were genetically stable over the project period.

## 4. Discussion

In line with our findings in this study large, complex buildings are known to be at higher risk of being colonised with *Legionella* [1,19]. Furthermore, the distribution between *L. pneumophila* sg1 and 2–14 was found to be 50/50 for the positive systems and in line with other findings for similar facility types [38]. 

It has been extensively reported that more than one *Legionella* species or sg can colonise a system [39]. Co-colonisation with low levels of *L. anisa* was found in four of the shower systems. However, only one *L. pneumophila* strain was detected within the sample period for 29 of the total 30 culture-positive municipal shower systems included in this study. In one of the larger nursing homes (H5) the detected strain varied from the initial to the current sampling round, see Table 2. It was not investigated when this shift in colonization strain occurred or if it was due to co-colonisation. There will always be a risk, in the chosen sampling scheme, that low abundance strains remain undetected or that only one strain from a bacterial community is selected for further characterisation. Other factors of importance are the failure to select the proper sampling points in a complex system and the sensitivity of the selected culture method.

The longitudinal mapping of all the municipal shower systems in Stavanger gave a unique opportunity to investigate the *Legionella* culture status in shower systems that were actively part of a risk management plan and subjected to *Legionella*-reducing measures when necessary. To our knowledge, this has not been conducted to this extent before in Norway. 

Following the LD case in 2007, a substantial effort was made by Stavanger municipality to develop and document the effect of their *Legionella* surveillance and prevention programme. In the current routines for *Legionella* prevention in the municipality, the *Legionella* species, serotype, and level of colonisation, together with the user groups, are taken into account when deciding the risk category of the system and, thus, what kind of actions and prevention measures to implement in order to reduce the risk [40]. 

During the project period, the municipality took many actions in culture-positive systems to decontaminate the systems. Although the technical status, such as correct temperature and removal of dead legs in the plumbing, was a focus for all the systems, systems with *L. pneumophila* sg1 were a priority when it comes to further measures, such as installation of filters, chemical disinfection, and continuous treatment. This focus has resulted in a reduction in both the number of positive sample points and in the bacteria level of *Legionella* in these systems. However, the results indicate that if a system becomes colonised with *Legionella* it is very difficult to fully decontaminate, even when extensive measures are taken. This is in line with other findings [41,42].

The surveillance and mapping of *L. pneumophila* in the municipal shower systems have resulted in a municipality overview of the occurrence of *Legionella* in their shower systems in a longitudinal perspective. Such information is valuable both in the routine *Legionella* management work and potentially in source investigation [43]. The data provide a systematic documentation for the *Legionella* management plan and document the effect of any preventive measures in their shower systems under their operating conditions. 

In summary, it has given the municipality a tool to focus their limited resources where it gives the highest value in terms of risk reduction for their inhabitants. Generally, the risk of LD is low in Norway, with 69 and 65 cases reported in 2018 and 2019, respectively, before the onset of the pandemic [44]. Excepting the initial case in 2007, no LD cases where a municipal shower system is the likely source of infection have been reported in Stavanger during the project period. The chosen approach is in line with the new European Water Directive. Here *Legionella* is listed as a waterborne pathogen to control, and a suggested approach is to focus resources and attention on controlling the *Legionella* variant that put most lives at risk, *L. pneumophila* [45,46].

Serotyping of *L. pneumophila* isolates gives indications of risk based on serotype but is less useful in source investigations. SBT has proven more useful in epidemiological studies and source investigations [47]. 

Previously, the genetic subtyping of *L. pneumophila* in epidemiology was performed mainly by pulse-field gel electrophoresis and SBT by Sanger sequencing [6,7,47,48,49]. More recently WGS has become the method of choice in outbreak investigations because of the increased resolution in characterisation of strains [9,10]. SBT revealed in this study that the *Legionella* culture-positive municipal shower systems in Stavanger were dominated by a few STs. Of the four main STs, three STs seem clustered quite closely together geographically while the fourth showed a looser cluster formation over a larger geographical area. *Legionella* is naturally occurring in low concentrations in fresh water, and the source of colonisation for a shower system is most likely the inlet water. The exact contamination source is not very likely identifiable, but due to the formation of geographical clusters, speculations can be made about the source being in the vicinity of these clusters. ST574 has so far only been reported in Norway [8]. ST574 differs from ST154 only in NeuA (8 SNPs), indicating that this is an ST that may have evolved in our region.

The initial genetic subtyping in this project was conducted by Sanger sequencing, and besides the two isolates within an ST with a differing sg, it was not possible to separate isolates within the same ST by serotyping and SBT combined. This means that if a patient or group of patients reports to have visited several of the municipal buildings culture-positive for a particular *L. pneumophila* ST, the source investigation team could point to all of these as a potential source of infection. In this study, it was investigated whether WGS could give a better resolution and, therefore, be a more useful tool for the municipality. The WGS showed that there were very few SNP differences between the initial and current isolates of the same ST, both over time in a system and between systems. The clusters of *L. pneumophila* sg1 isolates (ST1, ST154, and ST574) showed fewer differences than the sg2–14 cluster (ST1341), which is in line with Wells et al. [50]. 

In summary, the results support previous findings that neither sg nor ST alone gives the sufficient resolution to identify a source of infection or which isolates are more closely related [10,51]. The WGS data clearly provides better resolution. However, in this study, the low number of SNP differences between isolates of the same ST from different systems would still make it challenging to locate an exact source. The information from environmental surveillance WGS data, along with epidemiological data, can, however, give a good indication of the potential sources of LD among municipal shower systems and, therefore, can give a head start in source investigations, in line with recent experiences [43,52].

## 5. Conclusions

Mapping of the prevalence and strain characteristics of *L. pneumophila* in municipal shower systems over more than 10 years indicates that surveillance and targeted prevention measures, with a focus on high-risk systems, has been successful. A substantial decrease in the level of colonisation and the number of colonised systems was seen, with no known new cases of LD in Stavanger where the source was a municipal shower system. The WGS data showed that the *L. pneumophila* strains found in the municipal shower systems persist over years. The implementation of WGS for genomic typing has provided the municipality with a better tool and a detailed overview of the strain distribution that can be a valuable asset in future surveillance and source investigations.

## Figures and Tables

**Table 1:** Risk categories, criteria and sample frequencies according to the internal guidelines of Stavanger municipality.

Risk Category	Category Criteria	Sample Frequency as Listed in Municipal Guidelines (2012)	Sample Frequency in Project Period (2012–2021)
1	Known user groups with compromised immunity and/or detected *L. pneumophila* sg1 in the system	2 times a year; if consistent results, reduce to yearly.	Preventive measures and extensive sampling (minimum once a week for 4 weeks) to document the effect of the measures.
2	Open access to the public and/or detected *L. pneumophila* sg2–14 or *Legionella* spp. in the system	Yearly; if consistent results, reduce to every 2 years.	Yearly for the whole test period. Preventive measures and extensive sampling (minimum once a week for 4 weeks) to document the effect of the measures.
3	Restricted access and no *Legionella* spp. detected in the system	Yearly; if no *Legionella* detected, reduce to every 5 years.	Every 4 years for kindergartens. Every 3 years for smaller sports complexes with restricted access. Others as described in guidelines.

**Table 2:** Summary of the culture status and characterisation of *L. pneumophila* in culture-positive shower systems in Stavanger municipality. The shower systems are initially sorted into sg1 or sg2–14 and then by ST within the sg category.

			Initial Status			Current Status	
Code	Sg	ST	Positive/Total	CFU/mL	Treatment Measures	Positive/Total	CFU/mL
H5	1	1/574 ^1^	7/20	++	DL, T	1/22	+
K2	1	1	2/4	+	T	Not in use	
K3	1	1	3/5	+++	PC, T	Not in use	
SC4	1	1	5/5	+++ ^2^	DL, PC, T, F, CD, DF	0/8	+ ^3^
S8 *	1	59	6/6	+++	DL, T, CC, F	5/6	+
H3	1	154	2/12	+	DL, PC, T	0/12	ND
H4	1	154	6/17	++	DL, PC, T	1/17	+
S3	1	154	6/6	++	DL, PC, T, CD	0/9	ND
S4	1	154	5/6	+++	DL, PC, T, CD, F	2/10	+
S5	1	154	4/6	+++	DL, T	4/8	+
SC6	1	487	5/6	++	DL, PC, CD	0/6	ND
K1	1	574	2/2	+++	DL, T	Not in use	
O1	1	574	1/1	+	PC, T	Not in use	
SC3	1	574	5/11	+	DL, PC, CD	0/12	ND
SC5	1	574	7/7	++ ^2^	DL, PC, T, CD, F, CC	8/22	+
SC7	1	574	1/4	++	PC, T	0/15	ND
S6	3	87	8/8	++	T, F	7/8	++
S7	6	114	1/1	+	PC, T	2/3	+
SC9	4	1334	4/6	++	DL, T	Not in use	
SC2	3	1341	1/6	+	DL, T	2/6	+ ^4^
H1	6	1341	6/6	++ ^2^	DL, T	Not in use	
O2	6	1341	3/3	++	None	Not in use	
O3	6	1341	1/1	+	None	Not in use	
O4	6	1341	4/6	++	T	Not in use	
O5 **	2–14 ***	1341	2/2	+	None	2/2	++
S2	6	1341	5/5	+++	PC, T, F	5/5	++
SC1	6	1341	6/6	++++	DL, PC, T	8/10	++
SC8	6	1341	5/5	+++	PC, T, CD	Not in use	
H2	4	1361	3/3	++ ^2^	DL, T	Not in use	
S1	10	1361	4/4	+++	DL, PC, T, CD, F	4/4	++

Building types: SC = sports complexes, S = schools, H = Nursing homes, K = Kindergarten, O = Other. Positive/total shows the number of culture-positive sample points vs. the total number of sample points analysed in a system. Systems with 30% or more culture-positive sample points are indicated in bold. Culture (cfu/mL); ND: <5 cfu/mL (detection limit of method), +: 5–10 cfu/mL, ++: 10–100 cfu/mL, +++: 100–1000 cfu/mL, ++++: >1000 cfu/mL. Treatments: DL= Deadlegs removed, PC = Plumbing/heater changed, T = Thermal treatment, CC = Chemical continuous treatment, CD = Chemical discontinuous treatment, DF = Distal filter, F = automatic flushing. Buildings decontaminated or removed from the municipal portfolio are indicated by “not in use” under current status. ^1^ Initial ST1 and current ST574. ^2^ *L. anisa* occasionally detected in system in addition to *L. pneumophila,* ^3^ *Legionella* not detected in filtered shower water, only in tap water, ^4^ Numbers from 2020, increased level in 2021, not representative for the status the past 10 year. * Culture-positive first time 2017, ** Culture-positive first time 2016. *** determined by agglutination, specific sg not determined. In the current sampling round, serotypes were confirmed by agglutination only.

**Table 3:** Allelic profiles of *L. pneumophila* isolates from the municipal shower systems.

SBT	*flaA*	*pilE*	*asd*	*mip*	*mompS*	*proA*	*neuA*
154	11	14	16	16	15	13	2
574	11	14	16	16	15	13	11
1334	11	14	16	25	7	13	206
1	1	4	3	1	1	1	1
59	7	6	17	3	13	11	11
87	2	10	3	28	9	4	13
114	3	6	1	6	14	11	9
487	3	6	1	28	14	11	11
1341	3	13	1	3	14	9	207
1361	3	13	1	1	14	9	207

## Data Availability

All sequencing data were deposited in the European Nucleotide Archive under BioProject PRJE.B.50383.

## Supplementary Materials

The following are available online at https://www.mdpi.com/article/10.3390/microorganisms10030536/s1, Table S1: Short-read sequence names and accession numbers. Sequences ending with “1_LEGSURV” were sampled prior to 2021 (Initial), whereas sequences ending with “2_LEGSURV” were sampled in 2021 (Current), Table S2: Long-read sequence names and accession numbers. Sequences ending with “1_LEGSURV_L” were sampled prior to 2021 (Initial), whereas sequences ending with “2_LEGSURV_L” were sampled in 2021 (Current), Table S3: ST1 SNP matrix, Table S4: ST59 SNP matrix. Data were generated using snp-dists from the RedDog alignment file. Reference = BioSample SAMEA12579726, average coverage = 92.3%, Table S5: ST87 SNP matrix. Data were generated using snp-dists from the RedDog alignment file. Reference = BioSample SAMEA12579726, average coverage = 88.11%, Table S6: ST114 SNP matrix. Data were generated using snp-dists from the RedDog alignment file. Reference = BioSample SAMEA12579726, average coverage = 98.3%, Table S 7: ST154 SNP matrix, Table S 8: ST574 SNP matrix, Table S 9: ST1361 SNP matrix. Data were generated using snp-dists from the RedDog alignment file. Reference = BioSample SAMEA12579726, average coverage = 98.0%.
