# Supplementary material for: Legionella pneumophila in Municipal Shower Systems in Stavanger, Norway; A Longitudinal Surveillance Study Using Whole Genome Sequencing in Risk Management
Source: Microorganisms. 2022 Feb 28;10(3):536. doi: 10.3390/microorganisms10030536 (PMC8954845; doi:10.3390/microorganisms10030536)
Supplement: Supplementary file 1 [file microorganisms-10-00536-s001.zip › microorganisms-1599899-supplementary.pdf]

Table S 1: Short-read sequence names and accession numbers. Sequences ending with "1_LEGSURV" are sampled prior to 2021 (Initial), whereas sequences ending with "2_LEGSURV" are sampled in 2021 (Current).

Sample	BioProject	BioSample	Short-read run accession
SUH_H1_1_LEGSURV	PRJEB50383	SAMEA12579715	ERR8122854
SUH_H2_1_LEGSURV	PRJEB50383	SAMEA12579716	ERR8122855
SUH_H3_1_LEGSURV	PRJEB50383	SAMEA12579717	ERR8122856
SUH_H4_1_LEGSURV	PRJEB50383	SAMEA12579718	ERR8122857
SUH_H4_2_LEGSURV	PRJEB50383	SAMEA12579719	ERR8122858
SUH_H5_1_LEGSURV	PRJEB50383	SAMEA12579720	ERR8122859
SUH_H5_2_LEGSURV	PRJEB50383	SAMEA12579721	ERR8122860
SUH_K1_1_LEGSURV	PRJEB50383	SAMEA12579722	ERR8122861
SUH_K2_1_LEGSURV	PRJEB50383	SAMEA12579723	ERR8122862
SUH_K3_1_LEGSURV	PRJEB50383	SAMEA12579724	ERR8122863
SUH_O1_1_LEGSURV	PRJEB50383	SAMEA12579725	ERR8122864
SUH_O2_1_LEGSURV	PRJEB50383	SAMEA12579726	ERR8122865
SUH_O3_1_LEGSURV	PRJEB50383	SAMEA12579727	ERR8122866
SUH_O4_1_LEGSURV	PRJEB50383	SAMEA12579728	ERR8122867
SUH_O5_2_LEGSURV	PRJEB50383	SAMEA12579729	ERR8122868
SUH_S1_1_LEGSURV	PRJEB50383	SAMEA12579730	ERR8122869
SUH_S1_2_LEGSURV	PRJEB50383	SAMEA12579731	ERR8122870
SUH_S2_1_LEGSURV	PRJEB50383	SAMEA12579732	ERR8122871
SUH_S2_2_LEGSURV	PRJEB50383	SAMEA12579733	ERR8122872
SUH_S3_1_LEGSURV	PRJEB50383	SAMEA12579734	ERR8122873
SUH_S4_1_LEGSURV	PRJEB50383	SAMEA12579735	ERR8122874
SUH_S4_2_LEGSURV	PRJEB50383	SAMEA12579736	ERR8122875
SUH_S5_1_LEGSURV	PRJEB50383	SAMEA12579737	ERR8122876
SUH_S5_2_LEGSURV	PRJEB50383	SAMEA12579738	ERR8122877
SUH_S6_1_LEGSURV	PRJEB50383	SAMEA12579739	ERR8122878
SUH_S6_2_LEGSURV	PRJEB50383	SAMEA12579740	ERR8122879
SUH_S7_1_LEGSURV	PRJEB50383	SAMEA12579741	ERR8122880
SUH_S7_2_LEGSURV	PRJEB50383	SAMEA12579742	ERR8122881
SUH_S8_1_LEGSURV	PRJEB50383	SAMEA12579743	ERR8122882
SUH_S8_2_LEGSURV	PRJEB50383	SAMEA12579744	ERR8122883
SUH_SC1_1_LEGSURV	PRJEB50383	SAMEA12579745	ERR8122884
SUH_SC1_2_LEGSURV	PRJEB50383	SAMEA12579746	ERR8122885
SUH_SC2_1_LEGSURV	PRJEB50383	SAMEA12579747	ERR8122886
SUH_SC2_2_LEGSURV	PRJEB50383	SAMEA12579748	ERR8122887
SUH_SC3_1_LEGSURV	PRJEB50383	SAMEA12579749	ERR8122888
SUH_SC4_1_LEGSURV	PRJEB50383	SAMEA12579750	ERR8122889
SUH_SC4_2_LEGSURV	PRJEB50383	SAMEA12579751	ERR8122890
SUH_SC5_1_LEGSURV	PRJEB50383	SAMEA12579752	ERR8122891
SUH_SC5_2_LEGSURV	PRJEB50383	SAMEA12579753	ERR8122892
SUH_SC6_1_LEGSURV	PRJEB50383	SAMEA12579754	ERR8122893

SUH_SC7_1_LEGSURV	PRJEB50383	SAMEA12579755	ERR8122894
SUH_SC8_1_LEGSURV	PRJEB50383	SAMEA12579756	ERR8122895
SUH_SC9_1_LEGSURV	PRJEB50383	SAMEA12579757	ERR8122896

Table S 2: Long-read sequence names and accession numbers. Sequences ending with “1_LEGSURV_L” are sampled prior to 2021 (Initial), whereas sequences ending with “2_LEGSURV_L” are sampled in 2021 (Current).

Sample	BioProject	BioSample	Long-read run accession
SUH_O2_2_LEGSURV_L	PRJEB50383	SAMEA12579726	ERR8135371
SUH_S5_2_LEGSURV_L	PRJEB50383	SAMEA12579738	ERR8135372
SUH_SC4_2_LEGSURV_L	PRJEB50383	SAMEA12579751	ERR8135373
SUH_SC5_2_LEGSURV_L	PRJEB50383	SAMEA12579753	ERR8135374

Table S 3: ST1 SNP matrix.

	SUH_SC4_1_LEGSURV	SUH_SC4_2_LEGSURV	SUH_K2_1_LEGSURV	SUH_H5_1_LEGSURV	SUH_K3_1_LEGSURV
SUH_SC4_1_LEGSURV	0	0	5	5	5
SUH_SC4_2_LEGSURV	0	0	5	5	5
SUH_K2_1_LEGSURV	5	5	0	0	0
SUH_H5_1_LEGSURV	5	5	0	0	0
SUH_K3_1_LEGSURV	5	5	0	0	0

Table S 4: ST59 SNP matrix. Data generated using snp-dists from RedDog alignment file. Reference = BioSample SAMEA12579726, average coverage = 92.3%.

	Ref	SUH_S8_2_LEGSURV	SUH_S8_1_LEGSURV
Ref	0	80869	80870
SUH_S8_2_LEGSURV	80869	0	3
SUH_S8_1_LEGSURV	80870	3	0

Table S 5: ST87 SNP matrix. Data generated using snp-dists from RedDog alignment file. Reference = BioSample SAMEA12579726, average coverage = 88.11%.

	Ref	SUH_S6_1_LEGSURV	SUH_S6_2_LEGSURV
Ref	0	80532	80533
SUH_S6_1_LEGSURV	80532	0	1
SUH_S6_2_LEGSURV	80533	1	0

Table S 6: ST114 SNP matrix. Data generated using snp-dists from RedDog alignment file. Reference = BioSample SAMEA12579726, average coverage = 98.3%.

	Ref	SUH_S7_1_LEGSURV	SUH_S7_2_LEGSURV
Ref	0	13378	13377
SUH_S7_1_LEGSURV	13378	0	3
SUH_S7_2_LEGSURV	13377	3	0

Table S 7: ST154 SNP matrix.

	SUH_S5_1_LEGSURV	SUH_S3_1_LEGSURV	SUH_S5_2_LEGSURV	SUH_S4_1_LEGSURV	SUH_H3_1_LEGSURV	SUH_H4_1_LEGSURV	SUH_S4_2_LEGSURV	SUH_H4_2_LEGSURV
SUH_S5_1_LEGSURV	0	5	5	10	5	3	10	5
SUH_S3_1_LEGSURV	5	0	10	13	8	6	13	8
SUH_S5_2_LEGSURV	5	10	0	15	10	8	15	10
SUH_S4_1_LEGSURV	10	13	15	0	13	11	4	13
SUH_H3_1_LEGSURV	5	8	10	13	0	2	13	4
SUH_H4_1_LEGSURV	3	6	8	11	2	0	11	2
SUH_S4_2_LEGSURV	10	13	15	4	13	11	0	13
SUH_H4_2_LEGSURV	5	8	10	13	4	2	13	0

Table S 8: ST574 SNP matrix.

	SUH_SC5_2_LEGSURV	SUH_K1_1_LEGSURV	SUH_SC3_1_LEGSURV	SUH_O1_1_LEGSURV	SUH_SC7_1_LEGSURV	SUH_H5_2_LEGSURV	SUH_SC5_1_LEGSURV
SUH_SC5_2_LEGSURV	0	5	6	3	2	3	0
SUH_K1_1_LEGSURV	5	0	7	4	3	4	5

SUH_SC3_1_LEGSURV	6	7	0	5	4	5	6
SUH_O1_1_LEGSURV	3	4	5	0	1	2	3
SUH_SC7_1_LEGSURV	2	3	4	1	0	1	2
SUH_H5_2_LEGSURV	3	4	5	2	1	0	3
SUH_SC5_1_LEGSURV	0	5	6	3	2	3	0

Table S 9: ST1361 SNP matrix. Data generated using snp-dists from RedDog alignment file. Reference = BioSample SAMEA12579726, average coverage = 98.0%.

	Ref	SUH_S1_1_LEGSURV	SUH_S1_2_LEGSURV
Ref	0	2311	2313
SUH_S1_1_LEGSURV	2311	0	2
SUH_S1_2_LEGSURV	2313	2	0
